# Oxytetracycline and Florfenicol Concentrations in Food-Additive Premixes Authorised for Broiler Chickens: Assessing Degree of Agreement with Manufacturers Labelling

**DOI:** 10.3390/ani11061797

**Published:** 2021-06-16

**Authors:** Aldo Maddaleno, Matías Maturana, Ekaterina Pokrant, Betty San Martín, Javiera Cornejo

**Affiliations:** 1 Laboratory of Veterinary Pharmacology, Faculty of Veterinary and Animal Sciences, University of Chile, Santiago 8820808, Chile; amaddaleno@veterinaria.uchile.cl (A.M.); matias.maturana@ug.uchile.cl (M.M.); 2 Programa de Doctorado en Ciencias Silvoagropecuarias y Veterinarias, Campus Sur Universidad de Chile, Santa Rosa 11315, La Pintana, Santiago 8820808, Chile; katiavalerievna@ug.uchile.cl; 3 Food Safety Laboratory, Faculty of Veterinary and Animal Sciences, University of Chile, Santiago 8820808, Chile

**Keywords:** antimicrobial premixes, oxytetracycline, florfenicol, HPLC-MS/MS, UPLC-MS/MS

## Abstract

**Simple Summary:**

In this study, two analytical methodologies were developed for the analysis of florfenicol and oxytetracycline premixes, which were applied in the analysis of pharmaceutical formulations manufactured at the national level. These premixes were assessed, since florfenicol and oxytetracycline are widely used in poultry farming, as they are active against several types of bacteria and their cost/effectiveness ratio is quite attractive and are administered for the therapeutic treatment of bacterial infections. However, these premixes must actually contain the active ingredients at a concentration that matches what the manufacturer states on their labels. Otherwise, they will not reach plasma concentrations that are therapeutically effective. In this work we set forth to verify via LC-MS/MS whether three commercial formulations of oxytetracycline premixes, and two of florfenicol, effectively matched their labels or not. Interestingly, this methodology detected oxytetracycline at a higher concentration than expected for those formulations, whereas the concentrations of florfenicol were lower than the on-label statement for both formulations.

**Abstract:**

Antimicrobials premixes are the presentation of choice to administer drugs simultaneously to groups of animals in intensive husbandry systems that require treatment for pathologies of bacterial origin. Among the premixes available for use in poultry, florfenicol and oxytetracycline are commonly administered via food or water. However, their actual concentration in premixes must meet on-label statements to ensure plasma concentrations reach effective therapeutic levels. Hence, this work was designed for the purpose of verifying whether the concentration of antimicrobial present in five premixes matched their on-label statement. Three oxytetracycline premixes, and two of florfenicol, were analysed using a Xevo TQ-S micro UPLC-MS/MS, and an ABSciex API4000 HPLC-MS/MS, respectively. Analytical methodologies were implemented and validated, showing an R^2^ ≥ 0.99 for the calibration curves. Oxytetracycline was detected in these premixes at concentrations exceeding on-label statements by 13.28%, 21.54%, and 29.68%, whereas florfenicol concentrations detected in premixes were 13.06% and 14.75% lower than expected. Consequently, this work shows that the concentration of active ingredients that are present in commercial formulations effectively differ from those stated on premix labels, and it also highlights how unpredictable their range of variability might be. This must be addressed through solid and updated laws that guarantee an effective pharmaceutical product.

## 1. Introduction

By definition, and according to directive 2001/82 EC from the European Union, a veterinary drug is every drug or combination of chemicals presented as possessing healing or preventing properties in regard to animal diseases [1]. Using these drugs allows different options in terms of administration routes, which results in a variety of pharmaceutical presentations for the same active ingredient. In particular, antimicrobial premixes are the presentation of choice to administer drugs simultaneously to groups of animals in intensive husbandry systems. These premixes can be defined as ‘uniform mixtures of one or more micro-ingredients with a solvent and or a medium’, and they are used to facilitate that these micro-ingredients do spread homogeneously in a greater mixture [2]. In turn, this mixture should also readily integrate into either feed or water.

Use of antimicrobials has grown exponentially over the last few decades following the growth of livestock industry. According to estimations by Van Boeckel et al. [3], antimicrobial use in farm animals is projected to reach 200,235 metric tons by the year 2030. One of the main concerns of antimicrobial use in farm animals is the development of resistant microorganisms [3,4]. Today it is well known that the introduction of antimicrobial agents into production systems has created favourable conditions for the selection, spread, and persistence of antimicrobial resistant bacteria capable of causing infections in animals and humans [5].

As for the specific situation in the poultry industry, antimicrobial use in premixes has contributed to health and welfare improvements for broiler chickens and laying hens by means of reducing the incidence and mortality of bacterial infectious diseases, while also allowing for an easy and effective administration of these drugs [6]. Amphenicols and tetracyclines are two of the most widely used antimicrobials in our national industry of land animals, particularly in our poultry flocks. This preference becomes evident by the level of recorded sales for these drugs, reaching up to 6149.9 kg for amphenicols and 1631.9 kg of tetracyclines, mostly intended for an oral administration route [7].

Florfenicol (FF) is an antimicrobial from the phenicols class and is a structural analogue of thiamphenicol, which possesses a fluorine atom instead a hydroxyl group [8]. Phenicols inhibit protein synthesis in bacteria, binding to the 50S ribosomal subunit [9,10]. Florfenicol is widely used in veterinary field and poultry farming due to its activity on several bacteria, such as *Haemophilus* spp., *Salmonella typhi*, *Klebsiella pneumoniae*, *Staphylococcus aureus*, *Pasteurella multocida*, and *Escherichia coli* [11], hence this antimicrobial is an effective tool especially in the treatment of respiratory diseases. Concerning its pharmacokinetic characteristics, florfenicol exhibits high bioavailability, high distribution volume, and good tissue penetration, due to its relatively high lipid solubility [11,12,13,14].

Meanwhile, Oxytetracycline (OTC) is an antimicrobial from the tetracyclines class that is widely used in farm animals due to its pharmacologic characteristics, and adequate cost/effectiveness ratio, against pathologies caused by enterobacteria, *Pasteurella multocida*, *Mycoplasma gallisepticum*, *Escherichia coli*, *Avibacterium paragallinarum*, and *Mycoplasma synoviae* [15]. Both the pharmacokinetic and antimicrobial properties of this drug are influenced by its powerful chelating effect on metallic ions [16]. Consequently, its intestinal absorption might be noticeably altered if it administered along with feed, substances that increase stomach pH or contain bivalent or trivalent cations (as they may trigger the development of chelating complexes). Among the latter, these substances range from minerals such as calcium, magnesium, manganese, aluminium, zinc, iron, and bismuth, and even the water or feed use for diluting and administering oxytetracycline [8,17,18]. Additionally, production and storage conditions may alter the final concentration in the drug presentation [19].

In spite of the therapeutic effect of these drugs, they are not exempt of risks when used in farm animals. Several authors have pointed out that when antimicrobials are used therapeutically on poultry their residues can be transferred into meat and bones [20,21,22]. This situation represents a risk to any human population that could consume food sourced from these animals if either withdrawal periods or dosages stated on-label are not met, hence resulting in violations of maximum residue limits set for such drug [23,24].

Most antimicrobials used in livestock farming are administered via feed additives, which include a premix that must contain homogeneous concentrations of the active ingredient to ensure that concentrations in plasma are therapeutically effective. These concentrations must also follow the label statements to ensure not only that the active ingredient is released effectively but also avoiding any toxicity and side-effects, or the chance of therapeutic differences in the target animal populations [25,26,27]. Fernandez-González et al. [28] analysed OTC medicated feeds and premixes and determined a 30% decrease of these drug in the analysis of the medicated feed after the extrusion process. Furthermore, different studies implementing a methodology for the analysis and determination of veterinary drugs in premixes and medicated feeds by the high-performance liquid chromatography (HPLC) method are described [29,30,31].

Nevertheless, we are not aware of any study that might have assessed the purity declared of the active ingredient concentration stated on drug premixes’ labels, and it also seems that no independent studies have been published confirming the veracity of those concentration statements. In this regard, it is worth noting that the active ingredient on a pharmaceutical presentation can destabilise over time when stored or become altered when it is handled, resulting then in concentrations that do not met the levels stated on the label. For instance, several studies have attempted to assess the stability of vitamin premixes against environmental factors or the composition of different diets. According to the factors assessed in each study, the authors found significant differences not only in concentration but also in the bioavailability and composition of those premixes [32,33,34]. Any failure on the concentration stated on the label for the active ingredient, or if the formulation loses its homogeneity, may result in unintended consequences such as administering an incorrect dose, inducing intoxications, therapy failures, modifications of withdrawal periods, and heterogeneous therapeutic results [35]. This failures have been identified by several international organisms and described as current stewardship gaps [36]. Consequently, determining and verifying the concentration of active ingredients as they are stated on the label of premixes is of the utmost importance.

To this end, liquid chromatography coupled to mass spectrometry is a confirmatory technique that allows analysing a series of compounds in various biological matrices. This technique has been used by several researchers to identify unknown compounds, quantify known ones, and to know the structure and chemical properties of molecules [37]. The detection capability and soundness of this kind of instruments makes this the technique of choice for studies on the equivalence of chemical compounds that try to determine via spectroscopy the amount of antimicrobial drugs present in premixes [38,39].

Bearing in mind the aforementioned points, along with the fact that consumption of antimicrobial premixes in farm animals is an important therapeutic tool for the food industry [31], our work intended to verify that the amount of oxytetracycline and florfenicol present in different commercial premixes used for animal feeding actually matches the amount stated on their labels. The drug concentrations were determined using high and ultra-performance liquid chromatography–tandem mass spectrometry and then analysed to determine the stability of the formulation and the degree of agreement with the label statements of the manufacturers. Finally, we also assessed possible problems that might occur if the differences are significant and their impact on the efficacy of the therapy.

## 2. Materials and Methods

### 2.1. Solvents, Reagents, and Certified Standards

Contents of oxytetracycline (OTC) and florfenicol (FF) in national manufactured premixes were analysed using the following HPLC-grade solvents: methanol, formic acid, and ammonium formate, which were sourced from MERCK^®^ (Merck KGaA, Darmstadt, Germany). Oxytetracycline hydrochloride (CAS:6153-64-6) and florfenicol (CAS: 73231-34-2) certified standard was used for the implementation of analytical methodology, the in-house validation procedure and premix analysis. As internal standard a deuterated form of an amphenicol molecule (chloramphenicol D5) and a molecule from the tetracyclines class that show similar chemical characteristics to oxytetracycline (metacycline), were used. All certified-purity standards were manufactured by Dr. Ehrenstorfer™ (Wesel, Germany).

The solution stock and the working-solutions were prepared at a concentration of 1000 μg/g and 1000 μg/kg, respectively. Using as dilution solvent, mobile phase (in a proportion of 85:15 of mobile phase A and B)

The mobile phase for the oxytetracycline analysis was prepared at a ratio of 85:15 (*v*/*v*) of mobile phase A (2 mM ammonium formate plus 0.16% formic acid in water) and mobile phase B (2 mM ammonium formate plus 0.16% formic acid in methanol). The mobile phase for florfenicol analysis was prepared at a ratio of 85:15 (*v*/*v*) of mobile phase A, corresponding to acetic acid 0.1% in water (pH 3.7 ± 0.2), and mobile phase B, corresponding to acetic acid 0.05% in acetonitrile/water (50:50 *v*/*v*) (pH 3.7 ± 0.2).

### 2.2. Commercial Premixes of OTC and FF

Five commercial formulations for broiler chickens were selected from those authorised by the National Agriculture and Livestock Service of Chile (SAG, by its Spanish acronym). These formulations were medicated premixes containing OTC and FF intended for treatment of poultry infectious diseases. The validity period of the pharmaceutical formulations analysed was two years, from 2019 to 2021. Table 1 details the names assigned to each premix in this work, which were obscured with the purpose of preserving information confidentiality:

### 2.3. Sample Treatment

OTC and FF premixes were dissolved in mobile phase, according to each method. After samples were shaken for 15 min in a Multi Reax^®^ agitator (Heidolph Instruments GmbH and Co. KG, Schwabach, Germany) and sonicated by 15 min. Finally, the solution was diluted twice in mobile phase to ensure that levels within the calibration curve ranges. These dilutions were standardised at 50 μg/L and analysed in batches of 20 samples per product (120 samples in total) to reduce the analytical variability of calculations and thus increase the certainty of finding significant values. All analyses were performed in a single evaluation time from the same production batch.

Once the concentrations in every OTC and FF premix were measured, they were compared against the concentrations stated on their labels and the difference between both values was calculated and expressed as percentages.

### 2.4. Instrumental Analysis

The OTC and FF concentrations in the antimicrobial premixes were analysed using liquid chromatography coupled to mass spectrometry. OTC was analysed with a Xevo TQ-S micro. The ultra-performance liquid chromatography was composed of an Acquity pump, an Acquity FTN furnace, an Acquity FTN autosampler. All of these modules were sourced from Waters Technologies^®^ (Milford, MA, USA). In the case of FF, the analysis was through high-performance liquid chromatography-tandem mass spectrometry (HPLC-MS/MS), specifically an ABSciex API4000 triple quadrupole mass spectrometer. The LC system was composed by an Infinity 4000 Series (Agilent Technologies^®^, Santa Clara, CA, USA) pump, furnace, and autosampler.

### 2.5. Implementation and In-House Validation of the Analytical Method

The chromatographic conditions were implemented based on previous works by Gavilán et al. [40] on oxytetracycline and by Faulkner et al. [41] on florfenicol. The experimental parameters, as analytical column, injection volume, flow, and column temperature were adapted accordingly to the instruments and analytical conditions present in the Laboratory of Veterinary Pharmacology from the University of Chile (FARMAVET, by its Spanish acronym) (Table S1). All interest analytes were determined by their selected reaction monitoring (SRM) transition and their retention time.

In-house validation parameters for oxytetracycline and florfenicol were assessed independently, analysing the areas ratio found and following the recommendations from the Commission Decision 2002/657/EC [42]. An internal protocol was established, and the parameters assessed in this work for both analytical methods were: retention time, limit of detection (LOD), limit of quantification (LOQ), linearity of calibration curves, recovery, specificity, and precision through the analysis of repeatability and intra-laboratory reproducibility.

### 2.6. Statistical Analysis

The measurements batches for each drug formulation were analysed using a Wilcoxon Test to determine the statistical significance of the differences found between the observed concentration values and those stated on the labels. A difference probability of *p* > 0.05 was deemed statistically significant for all experiments. All these tests were performed using the statistical software Infostat^®^ [43].

## 3. Results

### 3.1. Implementation of HPLC-MS/MS and UPLC-MS/MS Chromatographic Conditions for Detection of Oxytetracycline or Florfenicol in Antimicrobial Premixes

The chromatographic conditions were implemented on the basis of ionisation parameters for the mass spectrometer (positive mode), determination of precursor ions and product ions for both analytes (based on their molecular weight as established in their analysis certificates). Table 2 details the mass spectrum conditions observed after optimising both instruments, whereas Table S1 lists the configuration of our chromatographic systems, and Table S2 lists the mobile phase gradients used in this work.

Once the chromatographic conditions were established, the retention times for OTC and FF chromatographic peaks were determined using six injections of certified standards at a concentration of 50 μg/kg. As detailed on Table 3, the retention times for all the analyses showed a standard deviation of less than 2.5%.

**Figure 1:**
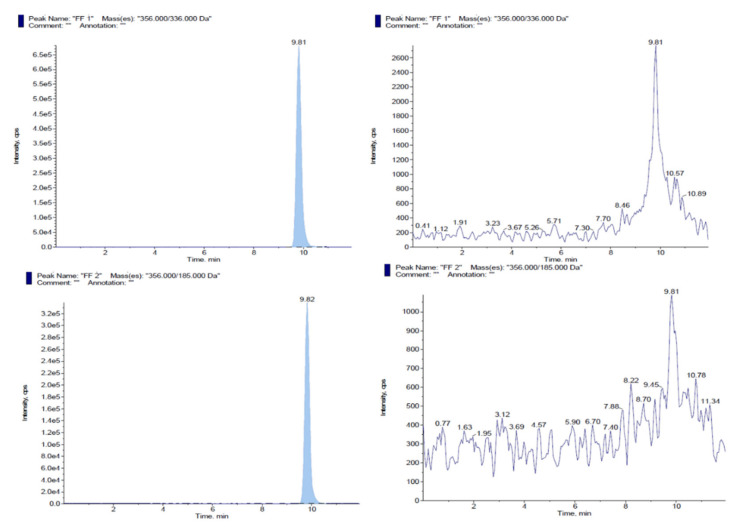
Chromatograms resulting from florfenicol standard solutions injection (**left**), as well as from the subsequent injection of a mobile phase solution (**right**). At the top ion fragment mass Q1 (356.00/336.00) and in the bottom ion fragment mass Q3 (356.00/185.00).

**Figure 2:**
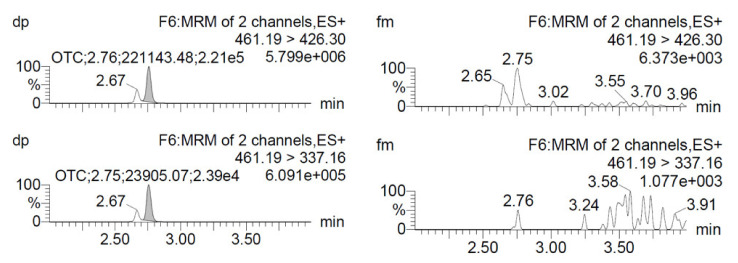
Chromatograms resulting from oxytetracycline standard solutions injection (**left**), as well as from the subsequent injection of a mobile phase solution (**right**). At the top ion fragment mass Q1 (461.19/426.30) and in the bottom ion fragment mass Q3 (461.19/337.16).

Additionally, the chance of recurrent contamination carried over from previous injections of each standard solution was determined by analysing chromatographically mobile phase injections performed after the injections of standard solutions. This is an important procedure because the occurrence of this kind of phenomena in highly sensitive instruments might result in chromatographic signals being added to a sample, which in turn leads to a faulty quantification of the certified purity in each formulation. Figure 1 and Figure 2 shows the chromatographic peaks observed in certified standard solutions for each analyte, as well as on mobile phases injected immediately after the standards. Importantly, the figure indicates that no chromatographic signals were observed after each injection that could interfere in the quantification of our samples.

### 3.2. In-House Validation of Analytical Methodologies

The specificity of the methods was assessed through the analysis of 20 analyses of dilution solvent for each method, which was compared with certified standard samples prepared at LOD concentrations, in order to compare the analytical signal at the retention times of the interest analytes. In both cases, there are no interferent at the retention times, indicating that both methods are specific for the analysed molecules.

In terms of the range of detection, we calculated the instrumental limit of detection (LOD) and limit of quantification (LOQ) of the method that were of 1 µg/kg and 1.022 µg/kg for OTC and 5 µg/kg and 5.451 µg/kg for FF, respectively. The signal/noise ratio for the detection and quantification limits was at least 3:1 and 10:1, for LOD and LOQ, respectively. To validate this parameter, 20 certified drug solution samples of 10 mL were prepared at 1 µg/kg of OTC and analysed by HPLC-MS/MS instrument to calculate the RSD of the obtained area/ratios from the OTC and MTC signal at the LOD concentration. The same procedure was realized to perform the LOD and LOQ of FF, using as internal standard CAF-D5. In each case, 1.64 times the SD obtained from the replicates was added to the defined LOD to obtain the LOQ concentration. The RSD of replicates was less than 25% in both cases.

To determine the linear response of concentrations versus instrumental detection, three calibration curves were performed at different times and using five equidistant concentrations for OTC and FF. To determine the linearity the R^2^ and the slope mean ± their standard deviation (SD) was calculated. For OTC, the calibrations curves were building at five concentrations at 10, 40, 80, 120, and 160 µg/kg of oxytetracycline. Moreover, for FF the calibration curves were building at 5, 40, 80, 120, and 160 µg/kg. These curves for each analyte showed a coefficient of determination (R^2^) > 0.99 (see Table 4).

To calculate the precision of both methods, six calibration curves of three points was prepared by different analyst in different days. For OTC, the building concentrations selected was 10, 80, and 160 µg/kg and for FF, concentrations of 5, 80, and 160 µg/kg. The precision for OTC was of 16.3%, and of 18.6% for FF.

As for recovery ratios, the calibration curves for this parameter were calculated in triplicate, using five equidistant points of OTC at concentrations of 5, 10, 20, 30, 40, and 50 µg/kg. Similarly, florfenicol curves were calculated also in triplicate, using concentrations of 20, 40, 60, 80, and 100 µg/kg. An internal standard was added to all analysed samples at a concentration of 1 µg/kg of metacycline or 4 µg/kg of chloramphenicol D5, respectively. Then, the standard recovery for each sample was calculated and the values observed were corrected according to the recovery observed for the internal standard.

### 3.3. Calculation of OTC and FF Concentrations in Antimicrobial Premixes

The antimicrobial concentration in each premix was quantified in mg/kg by replacing the areas ratio observed for each sample into the linear equation, which was calculated from a regression analysis of the calibration curves of pure standard solutions. The quantification of each analyte involved building curves at five concentrations (10, 40, 80, 120 and 160 µg/kg for OTC and 5, 40, 80, 120, and 160 µg/kg for FF. These curves were calculated to avoid extrapolating any data from the linear function at the time of assessing the concentrations for each drug, and they were expected to show an R^2^ ≥ 0.99.

**Figure 3:**
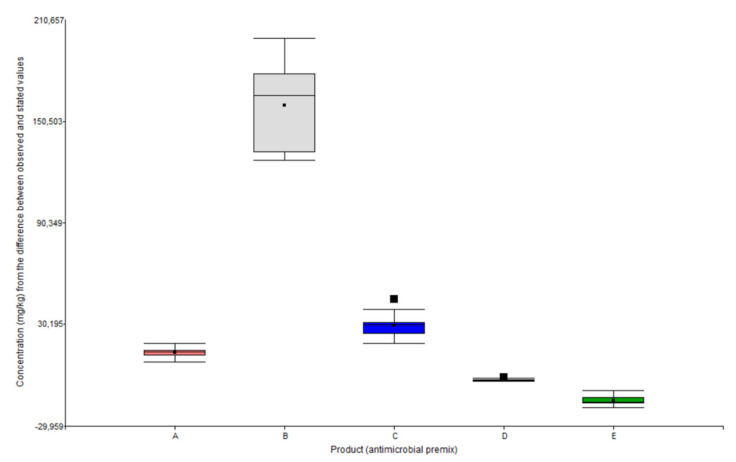
Box plot of the difference calculated between quantified concentrations (mg/kg) and stated concentrations, for oxytetracycline and florfenicol premixes.

The concentrations observed (mg/kg) in the samples (n = 20) from each premix were corrected by the recovery of the analytical method, and the comparison with the information stated on each product label has been detailed on Table 5. Additionally, Figure 3 shows box plots of each measurement batch in regard to concentration differences quantified (in mg/kg) v/s the values stated for each premix.

The concentration of oxytetracycline detected for all formulations exceeded the concentration stated on the label, averaging an additional 13.28, 21.54, and 29.68% of the active ingredient for products A, B, and C, respectively. The differences were statistically significant (*p* value < 0.0001) for all formulations according to the Wilcoxon Test, for a confidence level of 95% and a *p* value ≤ 0.05.

On the other hand, florfenicol concentrations detected in both formulations were below those stated on their labels. In particular, the concentration quantified for product D was −13.06% whereas for product E it was −14.75%. According to the statistical analysis, these differences were also statistically significant (*p* value ≤ 0.05).

## 4. Discussion

According to the results obtained in this study, concentrations of OTC and FF measured in different premixes were not fully consistent with the information stated on each product’s label. Oxytetracycline presented differences between the three formulations assessed that would lead to variations in the prescribed dose, affecting the withdrawal periods stablished for the pharmaceutical formulations. This might pose a risk, for both, the food production industry and consumers, because residues concentrations might exceed the MRLs set by authorities, even after meeting the withdrawal periods stated on the label of any of the formulations assessed in this study [44].

In the case of florfenicol, the concentrations of both formulations were below the percentage stated on the label (−13.06% and −14.75% for products D and E, respectively). These results imply that administering the therapy following the instructions provided by the manufacturer will increase the risk of therapy failure due to sub-dosing of the active ingredient. This would result in plasma concentrations that are below the targeted Minimum Inhibitory Concentration for the antimicrobial, as well as an increase in mortality rates within the affected farm and higher risk of resistance of pathogenic bacteria to antimicrobials.

Although the selection of antibiotic resistance has been studied mainly at concentrations above the MIC [45]. It has been observed that excessive concentrations of antibiotics cause selective pressure on clinically important bacteria, which means that only sensitive bacteria are killed, allowing antimicrobial-resistant bacteria to survive [46,47,48].

On the other hand, it has been described that at low antibiotic concentrations several cellular processes can be affected, increasing genetic variability, and altering the cellular behaviour of bacteria, although this is not yet well elucidated. Gullberg et al. [49], demonstrated, by highly sensitive competition experiments, that selection of resistant bacteria occurs at extremely low antibiotic concentrations.

In both cases, the therapy will fail when the composition of these formulations is not homogeneous, or if the actual concentration observed in them varies from what is stated on the label. Such failure can be ascribed to the uncertain plasma concentrations between individuals that follows from the ingestion of unknown doses, hence the actual behaviour of the active ingredient cannot be accurately predicted in all the animals. Not to mention the effects that are intrinsic to each diet and that can affect the absorption and stability of each active ingredient [50,51]. Consequently, the fact that these concentrations show so much variability demands that the concentrations stated on the label should be experimentally certified. It also implies that in the eventuality that alternative therapies do exist, they should be equivalent to ensure the efficacy of every dose administered to the animal population, hence preventing clinical and pathological differences between individuals [52]. However, in order to determine the therapeutic effect, considering the range of doses allowed according to manufacturer it is necessary to determine these effects through in vivo studies and, at the same time, to determine the bioavailability of these drugs by pharmacokinetic studies.

Recalculation of the oxytetracycline dose that should be administered involves weighing in less product for all three formulations (13.28%, 21.54%, and 29.68% less for formulations A, B, and C, respectively) than what is stated on their labels. Contrarily, florfenicol formulations would require weighing in more product to achieve a correct dose (14.75% and 13.06% more for formulations D and E, respectively) that comes close to that stated on the label.

This recalculation must be performed for both oxytetracycline and florfenicol, not only because this is the only way of ensuring a correct dose for the whole animal biomass but also because, as this work shows, ignoring such recalculation would also impede designing mathematical models that can accurately estimate the withdrawal periods for the desired Maximum Residue Limits, as well as the practical execution of the withdrawal procedure [53]. Notwithstanding, further comparative studies are required to assess other factors that may alter the purity of animal premixes over time, especially emphasising the stability of different active ingredients currently used in commercial antimicrobial premixes. In previous studies a degradation during the period of analysis was observed [54,55]. Nevertheless, in this study all premixes were maintained under light, temperature, and humidity conditions recommended by the manufacturer and the analysis was developed immediately after purchase of the premixes.

In the case of commercial formulations of florfenicol, two of the three formulations authorized for use in chickens were analysed. As for the analysis of the commercial formulations of oxytetracycline, three of the five formulations authorized by the agricultural and livestock service were analysed, since these were the formulations that were available on the market at the time of the study. All analysed samples were from the same production lot of each premix, since the analysis was performed in a single period of time. These are some of the limitations that should be recognized, although the results presented in this article, are interesting and lay the foundations for future drug stability and bioavailability studies.

## 5. Conclusions

This study shows that although the assessed premixes were homogeneous in terms of their composition, their observed concentrations did not match the concentrations stated on the package labelled by the manufacturer. Therefore, dose recalculation could be considered for antimicrobial drugs when therapy is unsuccessful to achieve desired drug concentrations in plasma. However, in order to do this, drug bioavailability should be evaluated or re-evaluated to ensure that animal products present limits of antimicrobial residues under the permitted residue limit after completion of the withdrawal period. This also indicates that a strict control is required over the manufacture process of these formulations, along with an update to current regulations overseeing the use and distribution of veterinary drugs.

## Figures and Tables

**Table 1:** Drug formulations analysed in this study.

Premix	Excipient	Active Product	Labelled Concentration	Manufacturer
Product A	100 g	10.78 g of Oxytetracycline dihydrate (equivalent to 10 g of Oxytetracycline base)	10%	1
Product B	100 g	80 g of Oxytetracycline hydrochloride (equivalent to 74.2 g of Oxytetracycline base)	80%	2
Product C	1 kg	100 g of Oxytetracycline	10%	2
Product D	100 mL	2 g of Florfenicol	2%	1
Product E	100 mL	10 g of Florfenicol	10%	2

**Table 2:** Mass fragmentation and instrument ionisation conditions.

Analyte	Polarity	Precursor Ion (*m*/*z*)	Product Ion (*m*/*z*)	DP ^5^ (volts)	EP ^6^ (volts)	CE ^7^ (volts)	CXP ^8^ (volts)
OTC ^1^	+	461.00	426.00	72.00	10.00	28.00	25.00
			381.00			36.00	22.00
MTC ^2^	+	443.17	426.15	20.00	15.00	20.00	30.00
FF ^3^	–	356.00	336.00	30.00	10.00	28.00	25.00
			185.00	30.00	10.00	28.00	25.00
CAF d5 ^4^	–	321.00	256.30	30.00	10.00	28.00	25.00

^1^ OTC: Oxytetracycline; ^2^ MTC: Metacycline; ^3^ FF: Florfenicol; ^4^ CAF d5: Chloramphenicol D5; ^5^ DP: Declustering potential; ^6^ EP: Entry potential; ^7^ CE: Collision energy; ^8^ CXP: Collision cell exit potential.

**Table 3:** Retention times observed for each analyte.

Analyte	Retention Time (min)						Mean (min)	SD ^5^	RSD ^6^ (%)
	1	2	3	4	5	6			
OTC ^1^ (461.19/426.3)	2.75	2.76	2.75	2.75	2.76	2.76	2.754	0.0054	0.21
OTC (461.19/337.16)	2.76	2.76	2.76	2.75	2.75	2.75	2.755	0.0055	0.20
TC-D6 ^2^ (443.17/426.24)	3.25	3.25	3.24	3.24	3.25	3.24	3.245	0.0055	0.17
FF ^3^ (356.00/336.00)	9.35	9.35	9.35	9.34	9.33	9.35	9.343	0.0082	0.09
FF (356.00/185.00)	9.34	9.34	9.34	9.34	9.31	9.35	9.337	0.0137	0.15
CAF-D5 ^4^ (321.00/256.80)	9.77	9.77	9.77	9.76	9.75	9.78	9.767	0.0103	0.11

^1^ OTC: Oxytetracycline; ^2^ TC-D6: tetracycline D6; ^3^ FF: Florfenicol; ^4^ CAF-D5: Chloramphenicol D5; ^5^ SD: Standard deviation; ^6^ RSD: Relative standard deviation.

**Table 4:** Analysis of calibration curves in certified standard solutions for oxytetracycline and florfenicol.

Analyte	Linearity		Recovery (%)	LOD ^4^ (μg/kg)	LOQ ^5^ (μg/kg)
	Mean R ^2,^* ± SD ^3^	Slope Mean ± SD			
OTC ^1^	0.9921 ± 0.003	0.4260 ± 0.028	98.6	1	1.022
FF ^2^	0.9957 ± 0.001	0.1771 ± 0.011	97.1	5	5.451

^1^ OTC: Oxytetracycline; ^2^ FF: Florfenicol; ^3^ SD: Standard deviation; ^4^ LOD; ^5^ LOQ; * Mean of Coefficient of determination.

**Table 5:** Actual concentrations observed for oxytetracycline and florfenicol premixes, and the differences found against the value stated on their label.

Premix	Active Ingredient	Labelled Concentrations (mg/kg)	Average Concentration ^3^ (mg/kg) ± SD ^4^	Difference of Concentration ^5^ (mg/kg)	Percentage of Difference ^6^ (%)
Product A	OTC ^1^	100,000	113,279.52 ± 2689.88	13,279.52	13.28
Product B	OTC	742,000	901,804.57 ± 24,297.15	159,804.57	21.54
Product C	OTC	100,000	129,678.38 ± 5987.27	29,678.38	29.68
Product D	FF ^2^	20,000	17,388.69 ± 627.02	−2611.31	−13.06
Product E	FF	100,000	85,247 ± 2484.82	−14,752.61	−14.75

^1^ OTC: Oxytetracycline; ^2^ FF: Florfenicol; ^3^ Average concentrations detected of the antimicrobial in the premix (mg/kg); ^4^ SD: Standard deviation; ^5^ Difference between the detected and stated concentrations; ^6^ Percentage of difference between the detected and stated concentrations.

## Data Availability

The data presented in this research are available in the article and supplementary material.

## Supplementary Materials

The following are available online at https://www.mdpi.com/article/10.3390/ani11061797/s1, Table S1: Configuration of chromatographic systems for Waters Xevo TQ-S micro and ABSciex API 4000 instruments., Table S2: Mobile phases gradient for the analysis of oxytetracycline, 4-epi-oxytetracycline, florfenicol, and florfenicol amine.

## References

[1] (2001). European Parliament and the Council of the European Union Directive 2001/82/EC of the European Parliament and of the Council of 6 November 2001 on the Community Code Relating to Veterinary Medicinal Products. Off. J. Eur. Union.

[2] Food and Agriculture Organization (2010). Good Practices for the Feed Industry: Implementing the Codex Alimentarius Code of Practice on Good Animal Feeding. FAO Animal Production and Health Manual.

[3] Van Boeckel T.P., Glennon E.E., Chen D., Gilbert M., Robinson T.P., Grenfell B.T., Levin S.A., Bonhoeffer S., Laxminarayan R. (2017). Reducing Antimicrobial Use in Food Animals. Science.

[4] Beyene T. (2015). Veterinary Drug Residues in Food-Animal Products: Its Risk Factors and Potential Effects on Public Health. J. Vet. Sci. Technol..

[5] Marshall B.M., Levy S.B. (2011). Food Animals and Antimicrobials: Impacts on Human Health. Clin. Microbiol. Rev..

[6] Venkitanarayanan K., Thakur S., Ricke S.C. (2019). Food Safety in Poultry Meat Production.

[7] (2016). Servicio Agrícola y Ganadero Declaración de Venta de Antimicrobianos. http://www.sag.cl/ambitos-de-accion/declaracion-de-venta-de-antimicrobianos.

[8] Bryskier A., André B. (2005). Antimicrobial Agents: Antibacterials and Antifungals.

[9] Ríos Insua A. (2005). Biodisponibilidad y Metabolismo de un Derivado Fluorado del Tianfenicol en Pollos Broiler. Doctoral Thesis.

[10] Calvo J., Martínez-Martínez L. (2009). Mecanismos de acción de los antimicrobianos. Enferm. Infecc. Microbiol. Clínica.

[11] Park B.-K., Lim J.-H., Kim M.-S., Hwang Y.-H., Yun H.-I. (2007). Pharmacokinetics of Florfenicol and Its Major Metabolite, Florfenicol Amine, in Rabbits. J. Vet. Pharmacol. Ther..

[12] (2007). American Academy of Veterinary Pharmacology and Therapeutics Veterinary Clinical Drug Information Monographs—Florfenicol. https://cdn.ymaws.com/www.aavpt.org/resource/resmgr/imported/florfenicol.pdf.

[13] Chang S.K., Davis J.L., Cheng C.N., Shien R.H., Hsieh M.K., Koh B.W., Chou C.C. (2010). Pharmacokinetics and Tissue Depletion of Florfenicol in Leghorn and Taiwan Native Chickens. J. Vet. Pharmacol. Ther..

[14] Patyra E., Kwiatek K. (2019). HPLC-DAD Analysis of Florfenicol and Thiamphenicol in Medicated Feedingstuffs. Food Addit. Contam. Part. A.

[15] Nelson M.L., Levy S.B. (2011). The History of the Tetracyclines: The History of the Tetracyclines. Ann. N. Y. Acad. Sci..

[16] Chopra I., Roberts M. (2001). Tetracycline Antibiotics: Mode of Action, Applications, Molecular Biology, and Epidemiology of Bacterial Resistance. Microbiol. Mol. Biol. Rev..

[17] MacDougall C., Chambers H. (2007). Antimicrobianos: Inhibidores de la síntesis de proteína y otros antibacterianos. Las Bases Farmacológicas de la Terapéutica.

[18] Ziółkowski H., Madej-Śmiechowska H., Grabowski T., Jaroszewski J.J., Maślanka T. (2019). Hard Water May Increase the Inhibitory Effect of Feed on the Oral Bioavailability of Oxytetracycline in Broiler Chickens. Pol. J. Vet. Sci..

[19] Yagoub Y.M.M., Abdoun S., Seri H.I. (2013). In-Use Stability Studies of Two Veterinary Medicinal Products: Albendazole and Oxytetracycline. Assiut Vet. Med. J..

[20] Abou-Raya Salah H., Shalaby Ali R., Salama Nadia A., Emam Wafaa H., Mehaya Fathy M. (2013). Effect of Ordinary Cooking Procedures on Tetracyclin Residues in Chicken Meat. J. Food Drug Anal..

[21] Alaboudi A., Basha E.A., Musallam I. (2013). Chlortetracycline and Sulfanilamide Residues in Table Eggs: Prevalence, Distribution between Yolk and White and Effect of Refrigeration and Heat Treatment. Food Control.

[22] Liu Y.-N., Pang M.-D., Xie X., Xie K.-Z., Cui L.-L., Gao Q., Liu J.-Y., Wang B., Zhang Y.-Y., Wang R. (2017). Residue Depletion of Amoxicillin and Its Major Metabolites in Eggs. J. Vet. Pharmacol. Ther..

[23] (2010). Commission Regulation (EU) No 37/2010 of 22 December 2009 on Pharmacologically Active Substances and Their Classification Regarding Maximum Residue Limits in Foodstuffs of Animal Origin. https://eur-lex.europa.eu/LexUriServ/LexUriServ.do?uri=OJ:L:2010:015:0001:0072:EN:PDF.

[24] Ziółkowski H., Jasiecka-Mikołajczyk A., Madej-Śmiechowska H., Janiuk J., Zygmuntowicz A., Dąbrowski M. (2020). Comparative Pharmacokinetics of Chlortetracycline, Tetracycline, Minocycline, and Tigecycline in Broiler Chickens. Poult. Sci..

[25] Lipinski C.A., Lombardo F., Dominy B.W., Feeney P.J. (1997). Experimental and Computational Approaches to Estimate Solubility and Permeability in Drug Discovery and Development Settings. Adv. Drug Deliv. Rev..

[26] Cheng Y., Samia A.C., Meyers J.D., Panagopoulos I., Fei B., Burda C. (2008). Highly Efficient Drug Delivery with Gold Nanoparticle Vectors for in Vivo Photodynamic Therapy of Cancer. J. Am. Chem. Soc..

[27] Biju V. (2014). Chemical Modifications and Bioconjugate Reactions of Nanomaterials for Sensing, Imaging, Drug Delivery and Therapy. Chem. Soc. Rev..

[28] Fernandez-González R., García-Falcón M.S., Simal-Gándara J. (2002). Quantitative Analysis for Oxytetracycline in Medicated Premixes and Feeds by Second-Derivative Synchronous Spectrofluorimetry. Anal. Chim. Acta.

[29] Krasucka D., Mitura A., Cybulski W., Kos K., Pietro W. (2010). Tiamulin Hydrogen Fumarate—Veterinary Uses and HPLC Method of Determination in Premixes and Medicated Feeding Stuffs. Acta Pol. Pharm..

[30] Song X., Xie J., Zhang M., Zhang Y., Li J., Huang Q., He L. (2018). Simultaneous Determination of Eight Cyclopolypeptide Antibiotics in Feed by High Performance Liquid Chromatography Coupled with Evaporation Light Scattering Detection. J. Chromatogr. B.

[31] Han J., Jiang D., Chen T., Jin W., Wu Z., Cui F. (2020). Simultaneous Determination of Olaquindox, Oxytetracycline and Chlorotetracycline in Feeds by High Performance Liquid Chromatography with Ultraviolet and Fluorescence Detection Adopting Online Synchronous Derivation and Separation. J. Chromatogr. B.

[32] Yang P., Wang H., Zhu M., Ma Y. (2019). Effects of Choline Chloride, Copper Sulfate and Zinc Oxide on Long-Term Stabilization of Microencapsulated Vitamins in Premixes for Weanling Piglets. Animals.

[33] Yang P., Wang H.K., Zhu M., Li L.X., Ma Y.X. (2020). Degradation Kinetics of Vitamins in Premixes for Pig: Effects of Choline, High Concentrations of Copper and Zinc, and Storage Time. Asian-Australas. J. Anim. Sci..

[34] Saensukjaroenphon M., Evans C.E., Paulk C.B., Gebhardt J.T., Woodworth J.C., Stark C.R., Bergstrom J.R., Jones C.K. (2020). Impact of Storage Conditions and Premix Type on Fat-Soluble Vitamin Stability1. Transl. Anim. Sci..

[35] Pauli G.F., Chen S.-N., Simmler C., Lankin D.C., Gödecke T., Jaki B.U., Friesen J.B., McAlpine J.B., Napolitano J.G. (2014). Importance of Purity Evaluation and the Potential of Quantitative 1H NMR as a Purity Assay: Miniperspective. J. Med. Chem..

[36] Patel S.J., Wellington M., Shah R.M., Ferreira M.J. (2020). Antibiotic Stewardship in Food-Producing Animals: Challenges, Progress, and Opportunities. Clin. Ther..

[37] Skoog D.A., Holler F.J., Crouch S.R. (2019). Espectrometría de masas molecular. Principios de Análisis Instrumental.

[38] Guo L., Chen Y., Zhang L., Yang W., He P. (2012). Development and Validation of a Liquid Chromatographic/Tandem Mass Spectrometric Method for Determination of Chlortetracycline, Oxytetracycline, Tetracycline, and Doxycycline in Animal Feeds. J. AOAC Int..

[39] Gavilán R.E., Nebot C., Patyra E., Vazquez B., Miranda J.M., Cepeda A. (2019). Determination of Florfenicol, Thiamfenicol and Chloramfenicol at Trace Levels in Animal Feed by HPLC–MS/MS. Antibiotics.

[40] Gavilán R.E., Nebot C., Veiga-Gómez M., Roca-Saavedra P., Vazquez Belda B., Franco C.M., Cepeda A. (2016). A Confirmatory Method Based on HPLC-MS/MS for the Detection and Quantification of Residue of Tetracyclines in Nonmedicated Feed. J. Anal. Methods Chem..

[41] Faulkner D., Cantley M., Walker M., Crooks S., Kennedy D., Elliott C. (2016). Evidence of Non-Extractable Florfenicol Residues: Development and Validation of a Confirmatory Method for Total Florfenicol Content in Kidney by UPLC-MS/MS. Food Addit. Contam. Part A.

[42] (2002). European Commission 2002/657/EC: Commission Decision of 12 August 2002 Implementing Council Directive 96/23/EC Concerning the Performance of Analytical Methods and the Interpretation of Results. Off. J. Eur. Union.

[43] (2020). Infostat® Version 2020I Update 04/30/2020. https://www.infostat.com.ar/.

[44] Chen J., Ying G.-G., Deng W.-J. (2019). Antibiotic Residues in Food: Extraction, Analysis, and Human Health Concerns. J. Agric. Food Chem..

[45] Hughes D., Andersson D.I. (2012). Selection of Resistance at Lethal and Non-Lethal Antibiotic Concentrations. Curr. Opin. Microbiol..

[46] Kristiansson E., Fick J., Janzon A., Grabic R., Rutgersson C., Weijdegård B., Söderström H., Larsson D.G.J. (2011). Pyrosequencing of Antibiotic-Contaminated River Sediments Reveals High Levels of Resistance and Gene Transfer Elements. PLoS ONE.

[47] Bengtsson-Palme J., Larsson D.G.J. (2016). Concentrations of Antibiotics Predicted to Select for Resistant Bacteria: Proposed Limits for Environmental Regulation. Environ. Int..

[48] Tello A., Austin B., Telfer T.C. (2012). Selective Pressure of Antibiotic Pollution on Bacteria of Importance to Public Health. Environ. Health Perspect..

[49] Gullberg E., Cao S., Berg O.G., Ilbäck C., Sandegren L., Hughes D., Andersson D.I. (2011). Selection of Resistant Bacteria at Very Low Antibiotic Concentrations. PLoS Pathog..

[50] Naidong W., Hua S., Roets E., Hoogmartens J. (2003). Assay and Purity Control of Tetracycline, Chlortetracycline and Oxytetracycline in Animal Feeds and Premixes by TLC Densitometry with Fluorescence Detection. J. Pharm. Biomed. Anal..

[51] Ziółkowski H., Grabowski T., Jasiecka A., Zuśka-Prot M., Barski D., Jaroszewski J.J. (2016). Pharmacokinetics of Oxytetracycline in Broiler Chickens Following Different Routes of Administration. Vet. J..

[52] Palacios-Arriaga J.M., Gutierrez-Pabello J.A., Chavez-Gris G., Hernandez-Castro R. (2000). Efficacy of Florfenicol Premix in Weaning Pigs Experimentally Infected with Actinobacillus Pleuropneumoniae. Rev. Latinoam. Microbiol..

[53] Chiţescu C.L., Nicolau A.I., Römkens P., Van Der Fels-Klerx H.J. (2014). Quantitative Modelling to Estimate the Transfer of Pharmaceuticals through the Food Production System. J. Environ. Sci. Health Part B.

[54] Bernabéu J.A., Camacho M.A., Gil-Alegre M.E., Torres-Suárez A.I. (2001). Procedure to Evaluate the Stability during Processing and Storage of a Medicated Premix and Medicated Farm Feed: Erythromycin Thiocyanate. J. Agric. Food Chem..

[55] Pérez-Lozano P., García-Montoya E., Orriols A., Miñarro M., Ticó J.R., Suñé-Negre J.M. (2006). Stability Evaluation of Amoxicillin in a Solid Premix Veterinary Formulation by Monitoring the Degradation Products through a New HPLC Analytical Method. J. Pharm. Biomed. Anal..

