# Supplementary material for: Oxytetracycline and Florfenicol Concentrations in Food-Additive Premixes Authorised for Broiler Chickens: Assessing Degree of Agreement with Manufacturers Labelling
Source: Animals (Basel). 2021 Jun 16;11(6):1797. doi: 10.3390/ani11061797 (PMC8234333; doi:10.3390/ani11061797)
Supplement: Supplementary file 1 [file animals-11-01797-s001.zip › animals-1142862-supplementary.pdf]

animals

Supplementary Materials

Oxytetracycline and Florfenicol Concentrations in Food-Additive Premixes Authorised for Broiler Chickens: Assessing degree of agreement with manufacturers labelling.

Aldo Maddaleno, Matías Maturana, Ekaterina Pokrant, Betty San Martín and Javiera Cornejo.

Table S1. Configuration of chromatographic systems for Waters Xevo TQ-S micro and ABSciex API 4000 instru-ments.

Configuration	Oxytetracycline analysis	Florfenicol Analysis
Detector	Waters XEVO TQ-S micro	ABSciex API 4000
Analytical column	Acquity ™ C 18 3.5 µm, 5 × 1.1 mm	Sunfire ™ C 18 3.5 µm, 150 × 2.1 mm
Injection volume (µL)	10	10
Oven temperature (°C)	36	35
Flow (mL/min)	0,200	0,250
Mobile phase A	Ammonium formate in water 0.0002 M and formic acid 0,16% in water	0.1% Formic acid in water
Mobile phase B	Ammonium formate in water 0.0002 M and formic acid 0,16% in methanol	0.1% Formic acid in methanol

Table S2. Mobile phases gradient for the analysis of oxytetracycline, 4-epi-oxytetracycline, florfenicol, and florfenicol amine.

Time (min)	Waters XEVO TQ-S micro		ABSciex API 4000	
	Phase A (%)	Phase B (%)	Phase A (%)	Phase B (%)
0	85	15	85	15
5	85	15	85	15
5.1	60	40	60	40
10	60	40	60	40
10.1	10	90	10	90
15	10	90	10	90
16	85	15	85	15
25	85	15	85	15
